# Case Report: Can Ruptured Aneurysms in the Hypoplastic and Plexiform Posterior Inferior Cerebellar Arteries Be Safely Occluded?

**DOI:** 10.3389/fneur.2022.904863

**Published:** 2022-06-24

**Authors:** Kun Hou, Jinlu Yu

**Affiliations:** Department of Neurosurgery, The First Hospital of Jilin University, Changchun, China

**Keywords:** posterior inferior cerebellar artery, hypoplastic, plexiform, aneurysm, endovascular treatment, subarachnoid hemorrhage

## Abstract

Aneurysms arising from the posterior inferior cerebellar artery (PICA) are not rare and may originate from the proximal or peripheral segment of the PICA. However, when the affected PICA is hypoplastic and plexiform, it is difficult to occlude the aneurysm without sacrificing the parent vessel, the PICA. This type of aneurysm is rare, and whether it is safe to occlude the aneurysm and the parent artery, in cases of a ruptured aneurysm of the hypoplastic and plexiform PICA, has not been adequately studied and is still open to debate. In this report, two patients with ruptured aneurysms in the hypoplastic and plexiform PICA were presented. Both patients were admitted to our hospital for subarachnoid hemorrhage. After team discussions between the neurosurgeons and neurointerventionalists, the aneurysm and parent PICA had to be occluded *via* endovascular treatment under general anesthesia. One of the patients developed postprocedural brainstem infarction and exhibited favorable recovery. The other patient died of pulmonary infection, although improvement in the postoperative state was observed. Although rare, aneurysms can originate from the hypoplastic and plexiform PICA. Occluding the aneurysm and hypoplastic parent PICA *via* endovascular treatment might be a reasonable option.

## Introduction

The posterior inferior cerebellar artery (PICA) is one of the three most important cerebellar arteries. Aneurysms arising from the PICA are not rare and may be located at its proximal or peripheral segment (1, 2). Generally, due to advancements in endovascular treatment (EVT), coiling the aneurysm while sparing the parent PICA is achievable because the involved PICA is often well-developed (3). However, when the affected PICA is hypoplastic and plexiform, it is difficult to occlude the aneurysm without sacrificing the parent PICA.

This type of aneurysm is rare, and whether it is safe to occlude the aneurysm and parent artery in cases of a ruptured aneurysm of the hypoplastic and plexiform PICA has not been adequately studied and is still open to debate (4, 5). In this report, we report 2 cases of ruptured aneurysms of a hypoplastic and plexiform PICA. Endovascular occlusion of the aneurysms and parent PICAs was performed, and no severe brainstem complications were encountered.

## Case Reports

### Case 1

**Figure 1:**
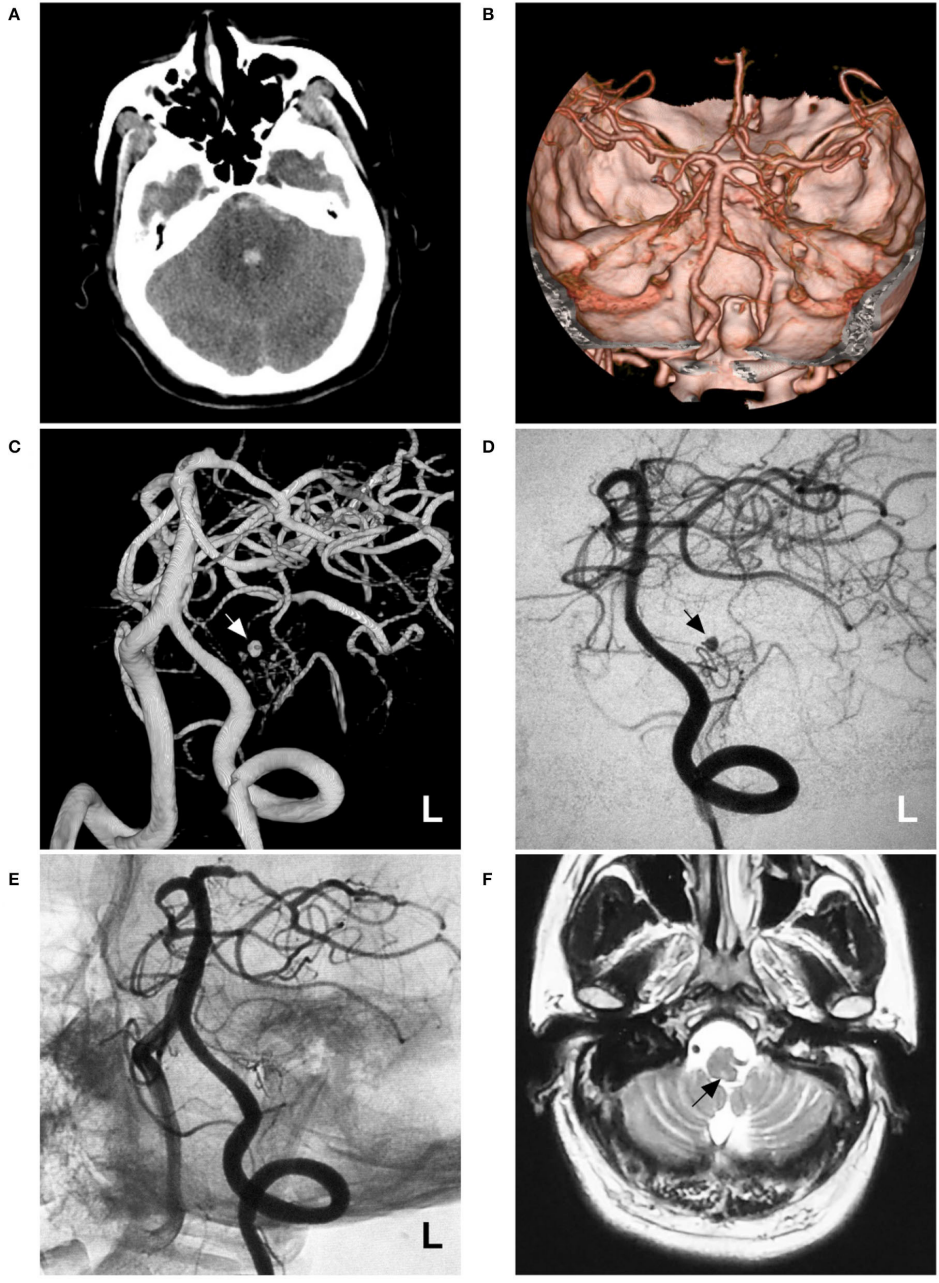
Imaging of case 1. **(A)** CT shows subarachnoid hemorrhage concentrated at the perimesencephalic cistern with involvement of the fourth ventricle. **(B)** CTA reveals no underlying vascular lesions. **(C,D)** Angiograms of the left VA show a pseudoaneurysm (arrow) located in the hypoplastic and plexiform PICA. **(C)** shows a three-dimensional angiogram, and **(D)** shows a two-dimensional angiogram. **(E)** Angiogram of the left VA shows that the aneurysm and parent PICA are cast with Onyx, and other branches are visualized. **(F)** Follow-up MRI scan shows a minor brainstem infarction (arrow). CT, computed tomography; CTA, CT angiography; L, left; MRI, magnetic resonance imaging; PICA, posterior inferior cerebellar artery; VA, vertebral artery.

A 53-year-old male suffered a sudden severe headache and vomited 4 h before admission. He was a Chinese patient of Han nationality who was healthy and denied having a history of chronic diseases. He had no history of drug abuse or surgical treatment of craniocerebral disease. Upon admission, a physical examination was performed, and the results were unremarkable, except for nuchal rigidity. Head CT showed subarachnoid hemorrhage (SAH) concentrated at the perimesencephalic cistern with involvement of the fourth ventricle (Figure 1A). CT angiography (CTA) revealed no underlying vascular lesions (Figure 1B). Catheter angiography revealed that the left PICA was hypoplastic and plexiform. Several small arteries originated from the left vertebral artery (VA) near the origin of the PICA. A pseudoaneurysm was located in the hypoplastic and plexiform PICA (Figures 1C,D). His family had no similar diseases.

Occlusion of the aneurysm and parent PICA *via* EVT was planned under general anesthesia. During the procedure, a Marathon microcatheter (Medtronic, Irvine, California, USA) was introduced into the PICA *via* the guidance of a microwire to access the aneurysm as much as possible. Then, the aneurysm and parent PICA were occluded with an Onyx liquid embolic system (Medtronic, Irvine, California, USA) (Figure 1E). Postoperatively, he showed no consciousness or movement disturbance. Mild hoarseness, dysphagia, and left central facial paralysis were noticed. The patient was discharged 1 week later. On follow-up one and a half years later, he recovered significantly, except for mild hoarseness. MRI only revealed the old brainstem infarction (Figure 1F).

### Case 2

**Figure 2:**
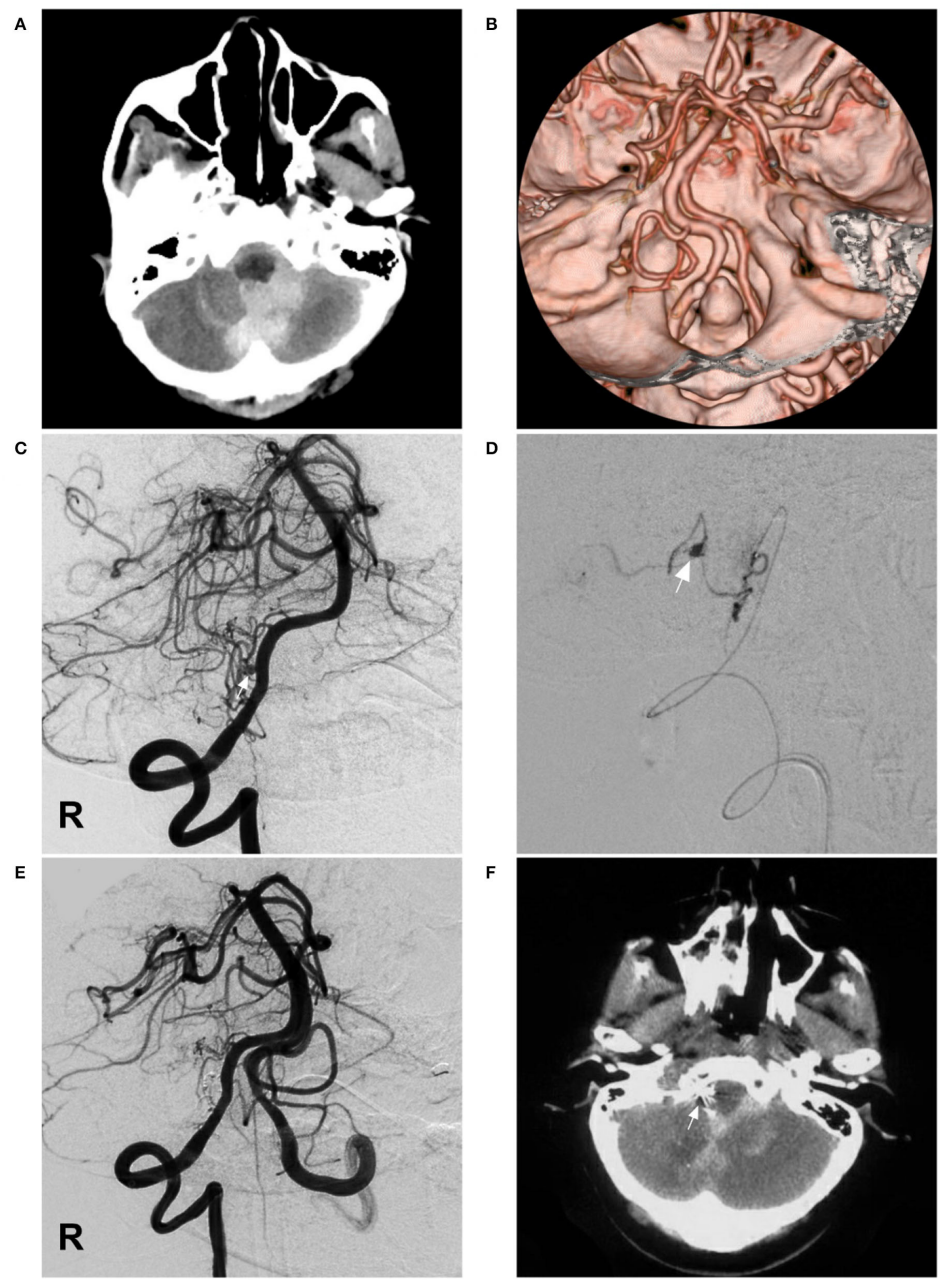
Imaging of case 2. **(A)** CT shows SAH concentrated at the cisterns around the brainstem and cerebellomedullary cistern. **(B)** CTA reveals no vascular abnormalities. **(C)** Angiogram of the right VA shows a pseudoaneurysm (arrow) located in the hypoplastic and plexiform PICA. **(D)** Superselective angiogram of the right PICA showing the pseudoaneurysm (arrow). **(E)** Angiogram of the VA shows that the aneurysm and parent PICA were cast with Onyx, and other branches are visualized. **(F)** Postoperative CT scan shows the location of casting Onyx (arrow) and partial resolution of the SAH. CT, computed tomography; CTA, CT angiography; PICA, posterior inferior cerebellar artery; R, right; SAH, subarachnoid hemorrhage; VA, vertebral artery.

A 60-year-old female suffered a sudden onset of headache and vomited 3 h before admission. She was a Chinese patient of Han nationality and had a 10-year history of diabetes, but she had no history of drug abuse or surgical treatment of craniocerebral diseases. On physical examination, she was drowsy. The strength of the four limbs was normal. Head CT showed SAH concentrated at the cisterns around the brainstem and cerebellomedullary cistern (Figure 2A). CTA revealed no vascular abnormalities that might be responsible for the SAH (Figure 2B). Catheter angiography showed that the right PICA was hypoplastic and plexiform. Multiple slim arteries originated from the right VA near the hypoplastic PICA. A pseudoaneurysm was detected in the plexiform PICA (Figures 2C,D). Her family had no similar disease.

Occlusion of the aneurysm and parent PICA *via* EVT was planned under general anesthesia. A Marathon microcatheter was advanced to the parent artery of the aneurysm *via* the guidance of a microwire to access the aneurysm. Then, the Onyx liquid embolic system was cast, successfully occluding the aneurysm and parent PICA (Figure 2E). The patient was stable postoperatively and regained consciousness 1 week later. A head CT scan performed 8 days later showed partial resolution of the SAH (Figure 2F). She was discharged to a local hospital for rehabilitation. A telephone follow-up revealed that she died of pulmonary infection 1 month later at the local hospital.

## Discussion

The PICA is a complex cerebellar artery (6, 7). In 80–95% of the cases, the PICA arises from the intracranial intradural VA. In 5–20% of the cases, the PICA has an extradural origin from the VA; rarely, the PICA may originate from the basilar artery (8). The PICA often begins in a single trunk, and in 2.5–6% of the cases, it begins from the VA as duplicate trunks (9). The PICA may be hypoplastic in 10–32% of the cases and absent on one side in 15–26% of the cases and on both sides in 2% of cases (10).

In hypoplastic PICA, the PICA may be plexiform and arise from the VA, consisting of multiple perforating arteries along the PICA course (8). Among these multiple perforating arteries, the strongest branch can be considered the hypoplastic PICA. These hypoplastic and plexiform PICAs mainly should perforate the brainstem rather than supply the posterior inferior facet of the cerebellum (11).

Aneurysms in hypoplastic and plexiform PICAs are rare entities. These aneurysms are dissecting aneurysms and can transform into pseudoaneurysms after rupture (1). They share the same pathophysiological mechanism with other intracranial perforator aneurysms, including the loss of internal elastic lamina induced by arteriosclerotic, hemodynamic, or inflammatory stress, such as perforating aneurysms in moyamoya disease (12, 13).

The ruptured pseudoaneurysms in hypoplastic PICA should be given aggressive treatment to avoid repeated rupture, including open surgery and EVT. The treatment with open surgery is challenging in the following two aspects: (a) it is difficult to identify the aneurysm intraoperatively, and (b) it is very hard to spare a hypoplastic PICA while occluding the aneurysm (14). Hence, EVT is a reasonable option. However, due to the narrow diameter of the PICA, it is challenging to embolize the aneurysm while sparing the parent PICA.

Therefore, occluding an aneurysm and hypoplastic parent PICA had to be performed. Although occluding an aneurysm and hypoplastic parent PICA is not technically difficult, it is unclear whether it is safe to occlude the hypoplastic PICA (1, 2).

Trivelato et al. reported that 14 patients harboring isolated dissecting aneurysms in the PICA with normal development were assigned to EVT, eight to selective coiling vs. six to parent artery occlusion (PAO). Both techniques were proven effective in preventing rebleeding; however, PAO was significantly associated with a higher risk of ischemic events (15). Based on the experience of the above study, two cases of this report can be tried to perform the occlusion of the hypoplastic and plexiform PICA.

In performing the PAO with the aneurysm in the hypoplastic and plexiform PICA, coiling was difficult to perform because the microcatheter of delivering coils was too thick and stiff, and it was difficult to go further in the hypoplastic PICA. In addition, the coil was too stiff and easily resulted in perforation of the vessel and aneurysm. Therefore, the Onyx liquid embolization system was a good choice. However, the drawback of Onyx is that it sacrifices too many normal vessels. In a previous report, for patients with basilar artery perforated aneurysms, occluding the parent vessel to the perforated aneurysm was acceptable, despite the risk of brainstem stroke in some patients (16).

However, the consequences of occluding a hypoplastic PICA have never been studied. Therefore, we tried to occlude the ruptured aneurysms in the hypoplastic and plexiform PICA because the supply region of the hypoplastic PICA might be compensated by the neighboring arteries and other cerebellar arteries. Although brainstem infarction can occur, we believe the benefits outweigh the risks. After informing the patients and families about surgical plans and risks, the occluding of ruptured aneurysms in hypoplastic and plexiform PICA was performed; fortunately, no severe complications occurred.

## Conclusion

Aneurysms in hypoplastic and plexiform PICA were rare. Sometimes, occluding the aneurysm and the hypoplastic parent PICA *via* EVT had to be the last resort. Because the supply region of the hypoplastic PICA can be compensated by the neighboring arteries, occluding the ruptured aneurysms in the hypoplastic and plexiform PICA may be feasible.

## Limitation

This is a report of two cases, and the conclusion of this study should be cautiously interpreted. In addition, no case of surgical treatment can be provided as a comparison with EVT, which was a limitation of the study.

## Data Availability Statement

The raw data supporting the conclusions of this article will be made available by the authors, without undue reservation.

## Ethics Statement

Written informed consent was obtained from the individual(s) for the publication of any potentially identifiable images or data included in this article.

## Author Contributions

JY designed the study and drafted the manuscript. KH collected the data. JY and KH confirm the authenticity of all the raw data. All authors have read and approved the final manuscript.

## Conflict of Interest

The authors declare that the research was conducted in the absence of any commercial or financial relationships that could be construed as a potential conflict of interest.

## Publisher's Note

All claims expressed in this article are solely those of the authors and do not necessarily represent those of their affiliated organizations, or those of the publisher, the editors and the reviewers. Any product that may be evaluated in this article, or claim that may be made by its manufacturer, is not guaranteed or endorsed by the publisher.
